# Effects of Salvianolic Acid B on Protein Expression in Human Umbilical Vein Endothelial Cells

**DOI:** 10.1155/2011/213050

**Published:** 2011-03-02

**Authors:** Tsong-Min Chang, Guey-Yueh Shi, Hua-Lin Wu, Chieh-Hsi Wu, Yan-Di Su, Hui-Lin Wang, Hsin-Yun Wen, Huey-Chun Huang

**Affiliations:** ^1^Department of Applied Cosmetology, Hungkuang University, Taichung 43302, Taiwan; ^2^Department of Biochemistry and Molecular Biology and Institute of Basic Medical Sciences, College of Medicine, National Cheng Kung University, Tainan 70101, Taiwan; ^3^School of Pharmacy, China Medical University, Taichung 40402, Taiwan; ^4^Department of Medical Laboratory Science and Biotechnology, College of Health Care, China Medical University, Taichung 40402, Taiwan

## Abstract

Salvianolic acid B (Sal B), a pure water-soluble compound extracted from Radix Salviae miltiorrhizae, has been reported to possess potential cardioprotective efficacy. To identify proteins or pathways by which Sal B might exert its protective activities on the cardiovascular system, two-dimensional gel electrophoresis-based comparative proteomics was performed, and proteins altered in their expression level after Sal B treatment were identified by MALDI-TOF MS/MS. Human umbilical vein endothelial cells (HUVECs) were incubated at Sal B concentrations that can be reached in human plasma by pharmacological intervention. Results indicated that caldesmon, an actin-stabilizing protein, was downregulated in Sal B-exposed HUVECs. Proteins that showed increased expression levels upon Sal B treatment were vimentin, T-complex protein 1, protein disulfide isomerase, tropomyosin alpha, heat shock protein beta-1, UBX domain-containing protein 1, alpha enolase, and peroxiredoxin-2. Additionally, Sal B leads to increased phosphorylation of nucleophosmin in a dose-dependent manner and promotes proliferation of HUVECs. We found that Sal B exhibited a coordinated regulation of enzymes and proteins involved in cytoskeletal reorganization, oxidative stress, and cell growth. Our investigation would provide understanding to the endothelium protection information of Sal B.

## 1. Introduction

Traditional Chinese medicine (TCM) has been widely practiced for thousands of years in China and Eastern Asia. TCM is now an integrated part of the healthcare system in that part of the world, and currently in China a strong push to modernize TCM through both scientific research and industrial development is underway. Radix Salviae miltiorrhizae (Danshen) has been used for the treatment of cardiovascular disorders and cerebrovascular diseases [1–4]. Salvianolic acid B (Sal B), a pure water-soluble compound extracted from Danshen, has been reported to possess many biological activities attributed to the whole-plant Danshen herb, such as sedative, antioxidant, hepatoprotective and antifibrogenic effects [5], inhibition of platelet aggregation, improvement of coronary microcirculation and cerebral blood flow [6], enhancement of angiogenic processes, and protection against injury to the heart and brain caused by ischemia reperfusion [7]. Following pretreatment with different dosages (0.0125–0.5 mg/mL) of Sal B for various times (2–36 h) followed by a 24 h incubation, Shi et al. demonstrated that Sal B upregulate the expression of tissue-type plasminogen activator (t-PA) and thrombomodulin (TM) and downregulate the expression of PAI-1 [8]. Our previous data also indicated that Sal B helps maintain the integrity of the endothelium by exerting anticoagulation and profibrinolytic effects in primary human umbilical vein endothelial cells (HUVECs).

In the past decades in Europe, the US, and other parts of the world, we have witnessed an increasing interest in the use of Sal B to formulate drugs, dietary supplements, and functional food products [9]. In the current study, we aimed to investigate the modes of actions of Sal B for the prevention of vascular diseases. A previous study reported an HPLC method for the determination of Sal B in rat plasma after oral administration of Danshen extract and the pharmacokinetics of Sal B were investigated. The results showed the concentration of Sal B in rat plasma over a concentration range of 10.8–259.4 *μ*g/mL [10]. The Sal B concentration of 125 *μ*g/mL that was predominantly used in this current study was intended to be clinically relevant. To identify proteins or pathways by which Sal B might exert its protective activities on the cardiovascular system, we surveyed global changes in proteins after Sal B treatment in HUVECs. We report here our initial findings of Sal B on the protein expression in HUVECs based on the well-established 2D gel/MALDI-TOF/TOF mass spectrometer analyses. Then, the differential expression of some proteins was confirmed by Western blot.

## 2. Materials and Methods

### 2.1. Cell Culture

HUVECs purchased from *Bioresource Collection and Research Centre, Hsinchu, Taiwan *were cultured in medium M-199 (Gibco BRL, Grand Island, NY, USA), supplemented with 10% fetal bovine serum (Gibco BRL, Grand Island, NY, USA), 1% endothelial cell growth supplement (Upstate Biotechnology, Lake Placid, NY, USA), 10 U/ml heparin, 100 U/ml penicillin, and 100 *μ*g/ml streptomycin (Gibco BRL, Grand Island, NY, USA). HUVECs of fourth to seventh passage were grown to confluence, then were trypsinized, suspended, and seeded into culture dishes or wells for experimental use.

### 2.2. Sample Preparation for 2-DE

Endothelial cells (1×10^6^ cells/dish) in 6 cm plate cultivated for 16 h were treated with 125 *μ*g/mL of Sal B (Ivy Fine Chemicals, NJ, USA) in M199 containing 0.1% FBS for 12 h. After additional 24 h incubation, cells were suspended in a lysis buffer (8 M urea, 4% CHAPS, 40 mM Tris, 12.5 mM DTT, and 0.5% carrier ampholytes). Cell lysate was sonicated on ice for approximately 2 min and centrifuged to remove debris. The protein concentration was assayed using a 2D quantification kit (Amersham Bioscience, Piscataway, NJ).

### 2.3. 2-DE

The first dimensional isoelectric focusing (IEF) was performed on precast 13 cm immobilized pH 4 to 7 nonlinear gradient (IPG) strip (Amersham Bioscience, Piscataway, NJ) at 20°C with a maximum current setting of 40 *μ*A/strip using an Amersham Pharmacia IPGphor IEF unit. Protein (100 *μ*g) was added to rehydration solution (7 M urea, 2 M thiourea, 4% CHAPS, 0.2% DTT, 0.5% Triton X-100, and 0.4% carrier ampholytes). Strips were rehydrated for 12 h, and then IEF was performed for a total of 45,000 Vh. Following IEF separation, the gel strip was first equilibrated for 15 min in the equilibration buffer consisting of 50 mM Tris-HCl pH 8.8, 6 M Urea, 30% glycerol, 2% SDS, and 2% DTE. Then the strip was equilibrated for another 15 min in the same equilibration buffer, except that DTE was replaced with 2.5% w/v iodoacetamide. Sodium dodecyl sulfate-polyacrylamide gel (SDS-PAGE) (10%) was applied for the second-dimension electrophoresis. After the second dimension, gel was silver stained according to manufacturer's instruction (Amersham Bioscience, Piscataway, NJ). Triplicate electrophoresis was performed for each pair of protein sample (control and Sal B treated) from three independent experiments to ensure reproducibility.

### 2.4. Image Acquisition and Analysis

The silver-stained 2-DE gel was digitally scanned as 2-DE image on the Typhoon 9200 fluorescence image scanner (amersham bioscience, Piscataway, NJ). Spot detection, quantification, and matching were managed using the ImageMaster software (amersham bioscience, Piscataway, NJ). The theoretical *M_r_* and p*I* values of the 2-DE markers were used to calibrate the *M*_*r*_ and p*I* of the protein spots in the 2-DE gels. Protein spots with twofold or more increased or decreased intensity between control and Sal B-treated groups with *P* < .05 (Student's *t*-test) were considered as significantly differentially expressed proteins. The protein spots were cut from the gels and used for MALDI-TOF MS/MS identification.

### 2.5. Tryptic In-Gel Digestion of 2-DE-Resolved Proteins

Protein spots were excised from the 2-DE gel and soaked in 25 mM ammonium bicarbonate (pH 8.5)/50 % acetonitrile at room temperature for 15 min. Following three washes with the above buffer, the gel pieces were dehydrated in 100% acetonitrile for 5 min and dried for 5 min in a speed-vac centrifuge. The gel pieces were swollen and crashed in 25 mM ammonium bicarbonate buffer containing 30 ng trypsin and incubated at 37°C for at least 16 hr. Peptide mixtures were extracted twice with 50% acetonitrile/5% trifluoroacetic acid (TFA) by occasional sonication and dried in a speed-vac concentrator. After dissolved in 0.1% TFA, the peptide mixtures were purified by using C_18_ Zip-Tip (Millipore Corporation, Billerica, MA, USA).

### 2.6. MALDI-TOF/TOF Mass Spectrometry and Protein Identification

Mass analyses were performed using a MALDI-TOF/TOF mass spectrometer (Ultraflex, Bruker Daltonics, Billerica, MA). Peptide mixtures were spotted onto the MALDI target plate using a saturated matrix solution of *α*-cyano-4-hydroxycinnamic acid (Sigma) in 80% acetonitrile/1% TFA. The instrument was externally calibrated with standard peptide mixtures and further adjusted with the lock mass feature using adrenocorticotropic hormone (ACTH) as the near-point calibration agent. Mass spectra were acquired for the mass range of 700–3000 Da and automatically processed by the Biotool Data Analysis software (Bruker Daltonics, Billerica, MA) and MASCOT software (Matrix Science, MA) for PMF searches against the SWISS-Prot database. The search parameters allowed for oxidation of methionine,* N*-terminal acetylation, and carboxyamidomethylation of cysteine. The criteria for positive identification of proteins were set as follows: (i) at least five matching peptide masses, (ii) 50 ppm or better mass accuracy, (iii) one incomplete cleavage tolerance, (iv) limiting the search to the human subset of the database and (v) molecular weight and P*I* of identified proteins should match the estimated values obtained from image analysis.

### 2.7. Electrophoresis and Immunoblot Analysis

HUVECs treated with Sal B or not were washed twice with cold phosphate-buffered saline (PBS), lysed in PBS containing 1% Nonidet P-40, 0.5% sodium deoxycholate, 0.1% sodium dodecyl sulfate (SDS), 5 *μ*g/mL aprotinin, 100 *μ*g/mL phenylmethylsulfonyl fluoride, 1 *μ*g/mL pepstatin A, and 1 mM ethylenediaminetetraacetic acid at 4°C for 20 min, and then disrupted with a needle. Total lysates were quantified using a micro-BCA kit (Thermo Fisher Scientific, Rockford, IL, USA). Proteins (50 *μ*g) were resolved by SDS-polyacrylamide gel electrophoresis and transferred electrophoretically to a PVDF filter. The nylon filter was blocked for 1 h in 5% fat-free milk in PBST buffer (PBS with 0. 05% Tween-20). After a brief wash in PBST buffer, the nylon filter was incubated overnight at 4°C with antibodies (mouse antihuman TM antibody (1 : 1000), mouse antihuman nucleophosmin antibody (1 : 1000), and mouse antihuman caldesmon antibody (1 : 1000), Santa Cruz Biotech, Santa Cruz, CA, USA), mouse antihuman vimentin antibody (1 : 1000), mouse antihuman T^199^ phosphonucleophosmin antibody (1 : 1000), and mouse antihuman *α*-tubulin antibody (1 : 2000) (Abcam, Cambridge Science Park, Cambridge, UK) diluted in PBST buffer. The primary antibody was removed, and the filter was washed extensively in PBST buffer. Subsequent incubation with goat anti-mouse antibody (1 : 10000) conjugated with horseradish peroxidase (Santa Cruz Biotech, Santa Cruz, CA, USA) proceeded at room temperature for 2 h. The filter was washed extensively in PBST buffer to remove the secondary antibody, and the blot was visualized with ECL reagent. Analysis of band densities was accomplished with ImageJ gel analysis software.

### 2.8. Proliferation Assay

HUVECs (7.5 × 10^4^ per well) were treated with or without Sal B (125 *μ*g/mL) for 12 h. Cells were quantified with a cell proliferation assay kit by using a 4,5-dimethylthiazol-3-carboxymethoxy-phenyl-4-sulfophenyl-tetrazolium (MTS) tetrazolium compound (Promega).

### 2.9. Statistical Analysis

All cellular experiments were performed at least 3 times. Data are expressed as the mean ± SD. Statistical analysis of the data was compared using the unpaired Student's *t*-test. Differences were considered as statistically significant at *P* < .05.

## 3. Results

### 3.1. 2-DE of Sal B-Treated HUVECs and Identification of Differentially Expressed Proteins

**Figure 1:**
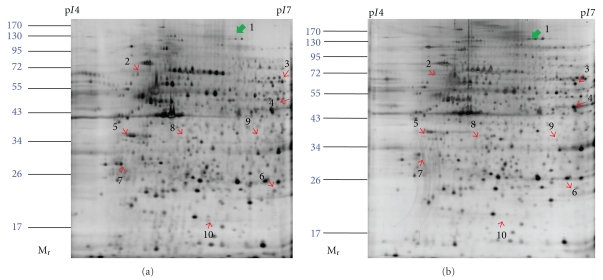
Silver-stained 2-DE gel of proteins extracted from Sal B-treated (a) and control (b) HUVECs. 100 *μ*g of total protein was subjected in linear IPG strips, with a pH range of 4 to 7, followed by 10% SDS-polyacrylamide gel electrophoresis. Molecular mass and p markers are indicated along the gels.

From our previous report, Sal B was shown to increase the fibrinolytic and anticoagulant potential of cultured HUVECs by upregulating the expression of t-PA and TM and by downregulating the expression of PAI-1 in a dose-dependent manner following a 12 h pretreatment [8]. To investigate the other target-related proteins of Sal B in HUVECs, protein profiles of control and Sal B-treated HUVECs were studied by comparative proteomic analysis. Representative 2-DE gel images for control and Sal B-treated HUVECs are shown in Figure 1. Up to 650 protein spots could be resolved from each gel. Nine upregulated protein spots and one downregulated protein spot in the Sal B-treated group compared with control were found. The ten differentially expressed proteins are indicated by arrows (Figure 1). The significance of the changes in protein abundance was calculated by Student's *t*-test. For all the selected spots, the *P* values between the control and Sal B-treated groups were less than  .05. The fold difference indicated the ratio of the intensity value of the Sal B-treated group relative to the value of control group. Notably, spots 3 and 8 were found only in the Sal B-treated group.

**Figure 2:**
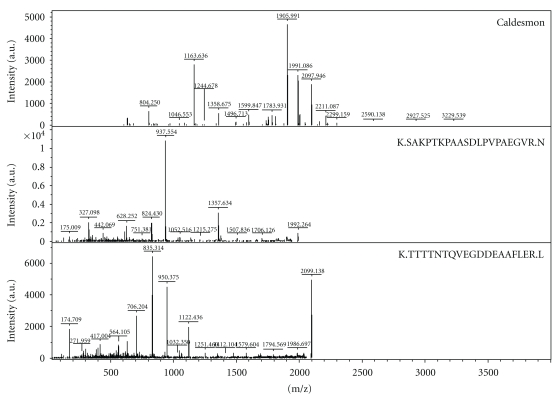
MALDI TOF/TOF MS/MS spectra of caldesmon isolated from a 2-DE gel. Out of 11 representative spectra, two peptides were matched and listed.

The differentially expressed protein spots were isolated from the 2-DE gel and subjected to trypsin digestion and identified using MALDI TOF/TOF mass spectrometry. The peptide mass peaks and peptide sequences were compared with those in the Swiss-Prot database. The MALDI-TOF MS/MS analysis result of caldesmon (CaD) is shown in Figure 2 as an example. The complete list of proteins identified unambiguously and the MALDI-TOF MS/MS analysis results are presented in Table 1. The Swiss-Prot accession number, theoretical molecular weight and p*I *of each protein spot, and the protein scores are also included. Furthermore, the reported localization and the biological function of the proteins are also listed. The altered proteins induced by Sal B treatment were categorized into several groups according to their putative/known functions. The major group of altered proteins was related to the cytoskeleton, including an increase in vimentin, T-complex protein 1*β*, and tropomyosin as well as the downregulation of caldesmon.

### 3.2. Western Blot Analysis

**Figure 3:**
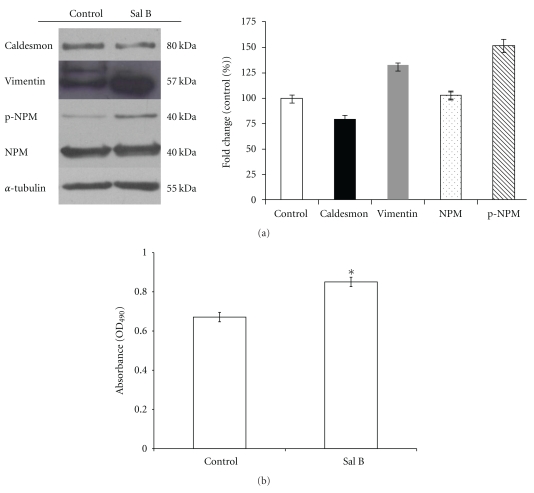
Sal B treatment decreased caldesmon expression and increased vimentin and phospho-NPM expression. (a) Cell lysates (50 *μ*g) were processed for Western blot analysis using monoclonal antibodies against TM and *α*-tubulin. The relative expression of caldesmon, vimentin, and NPM was quantified by densitometry and normalized to *α*-tubulin. Phospho-NPM was quantified by densitometry and normalized to the NPM protein. Control values were set to one. (b) Mitogenic effect of Sal B on HUVECs proliferation. HUVECs were seeded (7.5 × 10^4^ cells/well in 96-well tissue culture plate) and pretreated with Sal B for 12 h. The cells were washed and maintained in fresh EBM containing 0.25% FBS for additional 24 h incubation at 37°C. The cell viability was measured by the metabolic activity of viable cells with the Cell Proliferation Reagent WST-1. The values are presented as means of cell count obtained from six wells for each treatment. *P < .05 as compared with control as determined by analysis of variance followed by an unpaired Student's *t-*test.

The caldesmon, vimentin, and nucleophosmin (NPM) proteins were selected and subjected to Western blot analysis (Figure 3(a)). Compared with the control group, the level of caldesmon in the Sal B-treated group decreased, whereas the level of vimentin increased 1.4-fold after that treatment. Note that HUVECs expressed two isoforms of vimentin, a higher-molecular-mass form (experimental MW of 57 kDa) and a lower-molecular-mass form (experimental MW of 48 kDa), are both present in control and in Sal B-treated cells. The immunoblot data confirmed the 2-DE gel data. It has been found that protein expression levels of nucleophosmin are increased in 2D gel, whereas the Westernblot experiment using the anti-NPM antibody indicated that the total protein content of NPM did not change after Sal B treatment, since the differential protein spots showed electrophoretic mobility variation and moved to a more acidic position on the gel, which may be resulted from Sal B-mediated modification through post-translational phosphorylation of NPM. NPM was identified as a phosphoprotein, and several amino acid residues of this protein were identified as phosphorylation consensus sites. Among them, threonine 199 (Thr^199^) has been shown to be phosphorylated by cyclin E-cyclin-dependent kinase 2 (cyclin E/cdk2) during mitosis [11]. We therefore analyzed the phosphorylation of NPM by Western blot with an antibody specific for Thr^199^ phospho-NPM. Compared with the control group, the level of phospho-NPM in the Sal B-treated group increased to about 1.5-fold, which indicated that Sal B treatment induced progressive phosphorylation modification of NPM. We therefore examined the mitogenic effects of Sal B on HUVEC (Figure 3(b)). Sal B treatment resulted in an increased cell viability up to 25% of the control value.

**Figure 4:**
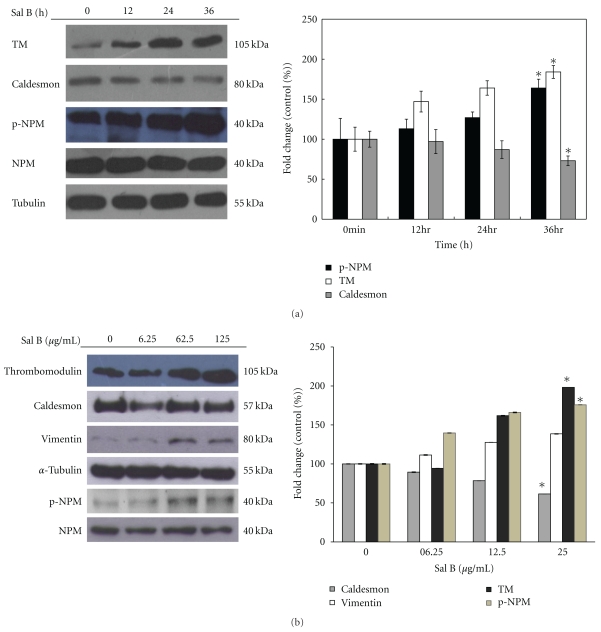
Western blot analysis of caldesmon, phospho-NPM, and vimentin expression after Sal B treatment. (a) HUVECs were pretreated with 125 *μ*g/mL of Sal B for 12 h. The cells were washed and maintained in fresh M199 medium for an additional 12 or 24 h incubation at 37°C. The expression levels of caldesmon, phospho-NPM, and vimentin were assayed. Total tubulin is shown as loading control. These data are representative results from three experiments. The relative amounts of caldesmon and vimentin, compared to total tubulin, and phospho-NPM normalized to the NPM were calculated, and the values represented the mean of triplicate experiments ± standard deviations. Control values were set to 100%. *P < .05 versus control. The bottom panels show that the densities of bands quantified by densitometry (b) HUVECs were pretreated with Sal B at the concentrations of 6.25, 12.5, or 25 *μ*g/mL for 12 h. Cells were then washed and maintained in fresh M199 for an additionally 24 h incubation at 37°C. Dose effects of Sal B on the expression of TM, caldesmon, vimentin, and phospho-NPM protein on HUVECs were demonstrated by Western blot. The tubulin or nucleophosmin was used as normalized control as described above. These data are representative results from three experiments. The bottom panels show the densities of bands analyzed by using the NIH software program ImageJ. *P < .05 versus control.

 We next evaluated the time course effects of Sal B-regulated expression of NPM, caldesmon, and TM in HUVECs. Expression levels of TM were elevated with increasing the Sal B incubation time at 12 h, which peaked by 24 h after stimulation (Figure 4(a)). To directly confirm the critical role of NPM, we measured the level of NPM phosphorylation at Thr^199^ in response to Sal B. As shown in Figure 4, treatment of HUVECs with Sal B resulted in a time-dependent phosphorylation of NPM at Thr^199^. Furthermore, increasing Sal B incubation time resulted in decreased expression of caldesmon to about 70% compared with the control. Moreover, the earlier studies on Sal B by Wang et al. showed that 10 *μ*M (7.18 *μ*g/mL) of Sal B can prevent epithelial-to-mesenchymal transition (EMT) triggered by TGF-*β*1 [12]. The dose-response relationships of TM, caldesmon, vimentin, and NPM, with respect to the low-dose ranges of Sal B treatment, were illustrated to confirm the protein profile change. HUVECs were pretreated with vehicle or increasing concentrations (6.25–25 *μ*g/mL) of Sal B for 12 h. When the concentration of Sal B was reduced to 6.25 *μ*g/mL, expression of TM was maintained at a constant level with minor decrease, whereas dose-dependent increase in TM was observed between the concentrations of 12.5 to 25 *μ*g/mL of Sal B. Additionally, after treated with Sal B at the concentrations of 6.25, 12.5, 25 *μ*g/mL, caldesmon levels were decreased proportionally to the Sal B exposure, which was about 45% of control level by administration of 25 *μ*g/mL of Sal B. It appeared that Sal B treatment caused a concentration-dependent enhancement of vimentin at concentrations ranging from 6.25 *μ*g/mL to 25 *μ*g/mL. We further investigated the effect of Sal B on the phosphorylation of NPM. Sal B began to induce NPM phosphorylation at 6.25 *μ*g/mL and continued in a dose-dependent manner (Figure 4(b)). Our results strongly suggest that Sal B treatment affects the protein profile and in a concentration-dependent manner on HUVECs.

## 4. Discussion

Danshen is one of the oldest herbs officially listed in the traditional Chinese Pharmacopoeia and is used in the treatment of myocardial infarction and other cardiac symptoms [3, 4, 13]. As a major constituent of Danshen, the action profile of Sal B on HUVECs has not been well documented. This study compared whole-cell proteins from HUVECs treated with Sal B to controls via a proteomic method that involved fractionation of proteins by 2-DE and identification of differentiated proteins by MALDI TOF MS/MS. We identified 10 protein spots that were involved in several biological pathways. Although we found the proteomics-based approach to be valuable in generating novel information, 2-DE bears some limitations in identifying low-abundance proteins. Due to these limitations, it is clear to understand that previously characteristic proteins such as TM and PAI-1 were not readily detected in our analysis.

Maintaining the integrity and function of the endothelial barrier is critical to vessel physiology. Those drugs and factors known to favorably influence endothelial cell function and promote angiogenesis have been shown to promote reendothelialization after vascular injury. We speculate that Sal B was able to induce an acceleration of the endothelial cell proliferation to refill an adequate cell density. The present investigation shows that Sal B induced phosphorylation of NPM. NPM has been reported to play a complex role in proliferation [14], apoptosis [15–17], and cell cycle progression [18]. It has also been postulated that NPM enhances proliferation by stimulating ribosome synthesis or DNA polymerase activity [19]. Previous studies have shown that human NPM is bound to unreplicated centrosomes in late G1 phase and undergoes phosphorylation by cyclin E/cdk2 at Thr^199^, prompting NPM's dissociation from the centrosome and subsequent duplication [20]. Other groups have provided evidence that induction of NPM protein expression is the critical limiting factor in NPM's ability to promote cell growth and proliferation [21, 22]. However, independent groups have demonstrated the roles of NPM, and its phosphorylation at Thr^199^ in the process of centrosome duplication does not account for the observed increase in cell growth and proliferation [23]. Salvianolic acid B stimulates the proliferation and tube formation of a murine simian virus resistance endothelial cell line through the sequential upregulation of the genes encoding MMP-2, VEGF, VEGF receptor 2, and Tie-1 [24]. To date, phosphorylation of NPM-Thr^199^ continues to be the subject of discussion and debate in the field. To investigate NPM's contribution to Sal B's protective effect, we tried to knockdown endogenous NPM, which resulted in HUVECs undergoing apoptosis (data not shown). Since NPM is considered as an antiapoptotic protein [17, 25], absence of NPM resulted in a dramatic loss of viability of HUVECs. Our results demonstrate that NPM plays an essential role in HUVECs and that Sal B may exert its potential protective effects by partially modulating NPM activity.

The low molecular weight isoform of caldesmon, l-CaD, is an actin-binding protein preferentially expressed in nonmuscle cells [26]. In several studies, l-CaD is described as a determinant factor in the organization and stabilization of the microfilament network in nonmuscle tissues and cells [27, 28]. Moreover, the cytostatic and proapoptotic potential of l-CaD revealed that it could regulate endothelial cell growth and survival via the modulation of cell shape and the cytoskeleton [29]. In our proteomics analysis, we found that Sal B decreased the expression of l-CaD, and this might affect cytoskeleton rearrangements, assembly of focal adhesions, and/or progression of HUVEC growth. This is consistent with previously finding that Sal B demonstrates enhancing effects on endothelial cell growth and differentiation. 

Besides caldesmon, intermediate filament vimentin is the other cytoskeleton protein which is modulated by Sal B. Endothelial cells contain an extensive interconnecting cytoplasmic network of vimentin. Vimentin is upregulated in migratory endothelial cells compared to nonmigrating endothelial cells [30]. Several physiological roles for vimentin have been proposed, including determination and maintenance of cell shape, transmission of mechanical stress, and the targeting of molecules between the nucleus and cytoplasm [31]. The cortex mesh of vascular endothelial cells consists of intertwined actin and vimentin [32]. It is noteworthy that *in vitro* vimentin synthesis is characteristic of proliferating cells [33]. The observed changes in vimentin expression suggested a possible role for Sal B in the transition of endothelial cells towards migrating and proliferating phenotypes. Interestingly, T-complex protein 1 (TCP-1) was also upregulated in Sal B-stimulated endothelial cells, and this protein is a member of the cytosolic chaperonin complex in eukaryotic cells [34]. TCP-1 containing chaperonins are nucleotide, actin, and tubulin binding proteins. Their expression, together with upregulation of other structural proteins such as vimentin and tropomyosin, suggest that changes in the concentration of these structural proteins may account for the proliferating/migration phenotype of endothelial cells under Sal B stimulation [28].

Peroxiredoxins, which are involved in intracellular redox balance and have stress-specific roles in stress resistance [35], are thioredoxin-dependent peroxide reductases localized either in the cytoplasm (PDX 1 and PDX 2) or mitochondria (PDX3) [36]. They represent a defense system against reactive oxygen species, and their peroxidase activity relies on thioredoxin. In addition, peroxiredoxin enzymes might participate in signaling cascades. This finding indicates the importance of antioxidant enzymes during the pretreatment of Sal B. These data suggested that the pharmacological potential of Sal B against pathological processes may be related to oxidative stress [37, 38]. 

In summary, Sal B treatment could change the levels of ten proteins that play important roles in protecting human endothelial cells via their effects on oxidative stress, cytoskeleton structure, and other processes. These observed changes in protein expression profiles translate into functional alternation and structure/morphological changes that might be necessary for the vascular protective effects of Sal B. Certainly, the identified proteins might not be the only targets of Sal B. Further studies in an effort to find other targets as well as functional characterization of proteins specifically regulated by Sal B will have an impact on treatments for improving of endothelial function.

## Figures and Tables

**Table 1:** Identified proteins that were changed in Sal B-treated HUVEC.

Spot no.	Protein identity	SwissProt no.	Relative spot intensity % (Sal B/control, 100%)	p*I*/*M_r_*	Mascot score	Cellular location	Molecular function
1	Caldesmon	Q05682	45%	5.5/93.3	81	Cytoskeleton	Actin binding, calmodulin binding, myosin binding, tropomyosin binding
2	Vimentin	P08670	227%	4.9/53.7	129	Cytoplasm intermediate filament	Protein binding, structural constituent of cytoskeleton
3	T-complex protein 1 subunit beta	P78371	Sal B only	6/57.8	223	Nucleus	ATP binding, unfolded protein binding
4	Protein disulfide-isomerase A3	P30101	260%	5.9/57.1	180	Endoplasmic reticulum melanosome	Cysteine-type endopeptidase activity, phospholipase C activity protein disulfide isomerase activity
5	Nucleophosmin	P06748	Sal B only	4.5/32.7	67	Nucleus, nucleoplasm, spindle pore cetrosome	
6	Heat shock protein beta-1	P04792	230%	6/22.8	135	Cell surface, cytoplasm nucleus	Identical protein binding
7	Tropomyosin alpha-3 chain	P06753	Only Sal B	4.5/32.9	85	Muscle thin filament tropomyosin	Actin binding
8	UBX domain-containing protein 1	Q04323	316%	5.1/33.4	153	Cytoplasm	ATPase binding, polyubiquitin binding
9	Alpha-enolase	P06733	237%	7.7/47.5	127	Nucleus, plasma membrane	Magnesium ion binding, serine-type endopeptidase activity, phosphopyruvate hydratase activity, transcription factor activity
10	Peroxiredoxin-2	P32119	258%	5.6/22	140	Cytoplasm	Thioredoxin peroxidase activity

## Abbreviations

SM: Radix Salviae miltiorrhizae
Sal B: Salvianolic acid B
HUVECs: Human umbilical vein endothelial cells
2-DE: Two-dimensional gel electrophoresis
MALDI-TOF MS/MS: Matrix-assisted laser desorption ionization time-over-flight/mass spectrometry
IPG: Immobilized pH gradient
IEF: Isoelectric focusing.

## References

[1] Feng P, Qin N, Qin Y (1999). Effect of composite salviae dropping pill on endothelin gene expression in circulating endothelial cells of patients with coronary heart disease. *Zhongguo Zhong Xi Yi Jie He Za Zhi*.

[2] Chun-sheng L, Hsiao-meng Y, Yun-hsiang H (1978). Radix salviae miltiorrhizae and Rhizoma ligustici wallichii in coronary heart disease. *Chinese Medical Journal*.

[3] Wu B, Liu M, Zhang S (2007). Dan Shen agents for acute ischaemic stroke. *Cochrane Database of Systematic Reviews*.

[4] Wu T, Ni J, Wei J (2008). Danshen (Chinese medicinal herb) preparations for acute myocardial infarction. *Cochrane Database of Systematic Reviews*.

[5] Lin YL, Wu CH, Luo MH (2006). In vitro protective effects of salvianolic acid B on primary hepatocytes and hepatic stellate cells. *Journal of Ethnopharmacology*.

[6] Tang MK, Ren DEC, Zhang JT, Du GH (2002). Effect of salvianolic acids from Radix Salviae miltiorrhizae on regional cerebral blood flow and platelet aggregation in rats. *Phytomedicine*.

[7] Chen YH, Du GH, Zhang JT (2000). Salvianolic acid B protects brain against injuries caused by ischemia-reperfusion in rats. *Acta Pharmacologica Sinica*.

[8] Shi CS, Huang HC, Wu HL (2007). Salvianolic acid B modulates hemostasis properties of human umbilical vein endothelial cells. *Thrombosis Research*.

[9] Chen J, Lee FSC, Li L, Yang B, Wang X (2007). Standardized extracts of Chinese medicinal herbs: case study of Danshen (Salvia miltiorrhiza Bunge). *Journal of Food and Drug Analysis*.

[10] Zhang J, Yu H, Sheng Y, Li L, Ye M, Guo D (2005). HPLC determination and pharmacokinetic studies of salvianolic acid B in rat plasma after oral administration of Radix Salviae Miltiorrhizae extract. *Biomedical Chromatography*.

[11] Tokuyama Y, Horn HF, Kawamura K, Tarapore P, Fukasawa K (2001). Specific phosphorylation of nucleophosmin on Thr^199^ by cyclin-dependent kinase 2-cyclin E and its role in centrosome duplication. *Journal of Biological Chemistry*.

[12] Wang QL, Tao YY, Yuan JL, Shen L, Liu CH (2010). Salvianolic acid B prevents epithelial-to-mesenchymal transition through the TGF-*β*1 signal transduction pathway in vivo and in vitro. *BMC Cell Biology*.

[13] Geng QX, Zhu XL, Zhang XH (2004). Effect of combined therapy of shenmai and compound danshen injection on myocardial reperfusion injury after percutaneous coronary intervention in patients with acute myocardial infarction. *Zhongguo Zhong Xi Yi Jie He Za Zhi*.

[14] Okuwaki M (2008). The structure and functions of NPM1/Nucleophsmin/B23, a multifunctional nucleolar acidic protein. *Journal of Biochemistry*.

[15] Ye K (2005). Nucleophosmin/B23, a multifunctional protein that can regulate apoptosis. *Cancer Biology and Therapy*.

[16] Frehlick LJ, Eirín-López JM, Ausió J (2007). New insights into the nucleophosmin/nucleoplasmin family of nuclear chaperones. *BioEssays*.

[17] Li J, Zhang X, Sejas DP, Bagby GC, Pang Q (2004). Hypoxia-induced nucleophosmin protects cell death through inhibition of p53. *Journal of Biological Chemistry*.

[18] Li J, Sejas DP, Rani R, Koretsky T, Bagby GC, Pang Q (2006). Nucleophosmin regulates cell cycle progression and stress response in hematopoietic stem/progenitor cells. *Journal of Biological Chemistry*.

[19] Lim MIJ, Wang XW (2006). Nucleophosmin and human cancer. *Cancer Detection and Prevention*.

[20] Okuda M, Horn HF, Tarapore P (2000). Nucleophosmin/B23 is a target of CDK2/cyclin E in centrosome duplication. *Cell*.

[21] Li Z, Boone D, Hann SR (2008). Nucleophosmin interacts directly with c-Myc and controls c-Myc-induced hyperproliferation and transformation. *Proceedings of the National Academy of Sciences of the United States of America*.

[22] Grisendi S, Bernardi R, Rossi M (2005). Role of nucleophosmin in embryonic development and tumorigenesis. *Nature*.

[23] Brady SN, Maggi LB, Winkeler CL (2009). Nucleophosmin protein expression level, but not threonine 198 phosphorylation, is essential in growth and proliferation. *Oncogene*.

[24] Lay IS, Chiu JH, Shiao MS, Lui WY, Wu CW (2003). Crude extract of Salvia miltiorrhiza and salvianolic acid B enhance in vitro angiogenesis in murine SVR endothelial cell line. *Planta Medica*.

[25] Jian Y, Gao Z, Sun J (2009). RNA aptamers interfering with nucleophosmin oligomerization induce apoptosis of cancer cells. *Oncogene*.

[26] Sobue K, Sellers JR (1991). Caldesmon, a novel regulatory protein in smooth muscle and nonmuscle actomyosin systems. *Journal of Biological Chemistry*.

[27] Mirzapoiazova T, Kolosova IA, Romer L, Garcia JGN, Verin AD (2005). The role of caldesmon in the regulation of endothelial cytoskeleton and migration. *Journal of Cellular Physiology*.

[28] Lay IS, Hsieh CC, Chiu JH, Shiao MS, Lui WY, Wu CW (2003). Salvianolic acid B enhances in vitro angiogenesis and improves skin flap survival in Sprague-Dawley rats. *Journal of Surgical Research*.

[29] Numaguchi Y, Huang S, Polte TR, Eichler GS, Wang N, Ingber DE (2003). Caldesmon-dependent switching between capillary endothelial cell growth and apoptosis through modulation of cell shape and contractility. *Angiogenesis*.

[30] McInroy L, Määttä A (2007). Down-regulation of vimentin expression inhibits carcinoma cell migration and adhesion. *Biochemical and Biophysical Research Communications*.

[31] Ivaska J, Pallari HM, Nevo J, Eriksson JE (2007). Novel functions of vimentin in cell adhesion, migration, and signaling. *Experimental Cell Research*.

[32] Pesen D, Hoh JH (2005). Micromechanical architecture of the endothelial cell cortex. *Biophysical Journal*.

[33] Wang N, Stamenović D (2000). Contribution of intermediate filaments to cell stiffness, stiffening, and growth. *American Journal of Physiology*.

[34] Kubota H, Hynes G, Willison K (1995). The chaperonin containing *t*-complex polypeptide 1 (TCP-1)—multisubunit machinery assisting in protein folding and assembly in the eukaryotic cytosol. *European Journal of Biochemistry*.

[35] Vang S, Corydon TJ, Børglum AD (2005). Actin mutations in hypertrophic and dilated cardiomyopathy cause inefficient protein folding and perturbed filament formation. *FEBS Journal*.

[36] Kang SW, Chae HZ, Seo MS, Kim K, Baines IC, Rhee SG (1998). Mammalian peroxiredoxin isoforms can reduce hydrogen peroxide generatedin response to growth factors and tumor necrosis factor-*α*. *Journal of Biological Chemistry*.

[37] Zhao GR, Zhang HM, Ye TX (2008). Characterization of the radical scavenging and antioxidant activities of danshensu and salvianolic acid B. *Food and Chemical Toxicology*.

[38] Chan K, Chui SH, Wong DYL, Ha WY, Chan CL, Wong RNS (2004). Protective effects of Danshensu from the aqueous extract of Salvia miltiorrhiza (Danshen) against homocysteine-induced endothelial dysfunction. *Life Sciences*.

